# Search for functional amyloid structures in chicken and fruit fly female reproductive cells

**DOI:** 10.1080/19336896.2020.1859439

**Published:** 2020-12-10

**Authors:** V. A. Siniukova, J. V. Sopova, S. A. Galkina, A. P. Galkin

**Affiliations:** a St. Petersburg Branch, Vavilov Institute of General Genetics, St. Petersburg, Russian Federation; b Laboratory of Amyloid Biology, St. Petersburg State University, St. Petersburg, Russian Federation; c Department of Genetics and Biotechnology, St. Petersburg State University, St. Petersburg, Russian Federation

**Keywords:** Functional amyloids, chicken, oocyte, fruit fly, eggshell

## Abstract

We conducted a cytological search for amyloid structures in female reproductive cells of *Gallus gallus domesticus* and *Drosophila melanogaster*. We have shown that the amyloid-specific dye, Thioflavin S, but not Congo red, stains some cytoplasmic and even nuclear structures in chicken ovaries. In fruit fly eggs both Thioflavin S and Congo red specifically stain eggshell structures such as micropyle, dorsal appendages and pillars. Moreover, these structures, when stained with Congo red, demonstrate birefringence in polarized light, which is a characteristic feature of all classical amyloids. Our data show that female reproductive cells during evolution began to use amyloid fibrils to form various functional structures necessary for development under certain environmental conditions.

## Introduction

Amyloid proteins traditionally associated with incurable diseases can perform vital functions in a variety of organisms. Amyloid fibrils have a cross-beta structure, bind Congo red and demonstrate apple-green ‘birefringence’ after such staining. They exhibit unique mechanical strength, elasticity, and resistance to a variety of agents. These properties of amyloid fibrils make them an irreplaceable structural component of protozoa and multicellular organisms. In particular, amyloid fibrils form a biofilm that allows bacteria to bind to the substrate [1], are a component of RNP particles in mammalian and insect brain neurons [2,3] and form deposits necessary for the synthesis of melanin [4]. Amyloid structures are found in eggshells of silk moth [5], fish, and mammalian oocytes [6,7]. Moreover, amyloid fibrils are a constitutive component of mammalian sperm acrosome [8]. It is assumed that the interaction of amyloid proteins of oocytes and sperm is an important factor of the acrosomal reaction during fertilization [8].

Here we performed a search for amyloid-like structures in *Gallus gallus domesticus* oocytes and in *Drosophila melanogaster* eggshell also called chorion. Chicken and fruit fly are important model organisms for developmental and genetic research. They are very convenient objects for the detection and study of functional amyloids. The results obtained do not allow us to draw final conclusions about the presence of amyloid structures in chicken oocytes. However, we showed that the specific structures of the fruit fly chorion, controlling fertilization and respiration, contain amyloid proteins.

## Results

**Figure 1.**
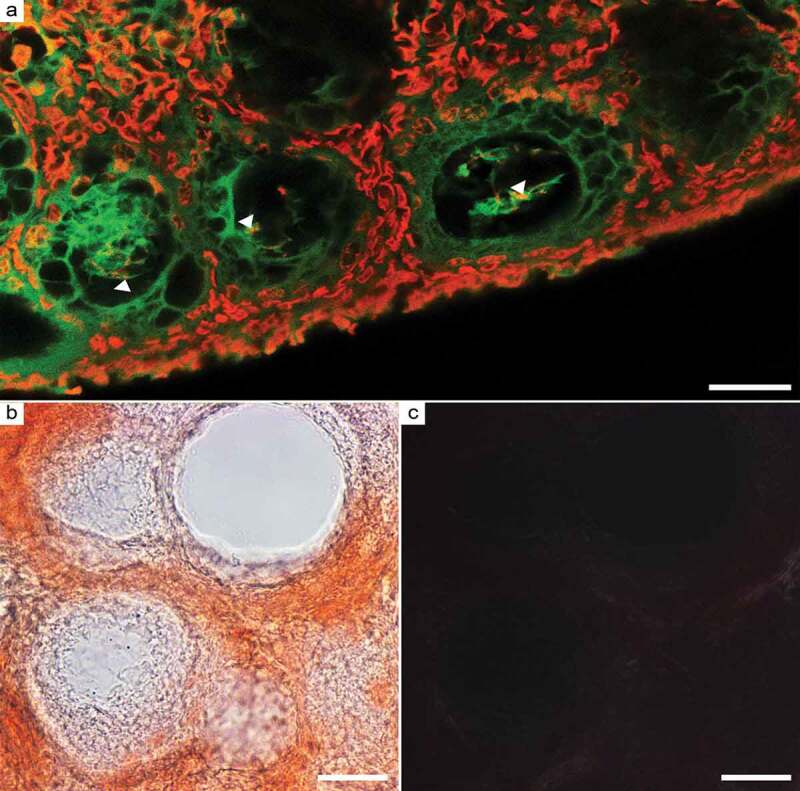
Chicken ovary cryosections stained with amyloid-specific dyes. (a) Thioflavin S staining (green fluorescence). Chromatin in nuclei was counterstained with TO-PRO-3 (red fluorescence). Areas of Thioflavin S and TO-PRO-3 colocalization (yellow) in oocyte nuclei are indicated by arrowheads. (b, c) Congo red staining visualized in brightfield (b) and between cross polarizers (c). Scale bars 25 µm

In order to assess the presence of amyloid-like structures in chicken oocytes, cryosections of ovaries of White Leghorn laying hens were prepared and stained with amyloid-specific dyes Thioflavin S and Congo red. Thioflavin S positive staining was detected in follicular cells and in cytoplasm of oocytes at the early stages of development (Figure 1(a)). Moreover, we found that Thioflavin S stains some nuclear chromatin-associated structures (Figure 1(a)). Note that no staining is detected in oocytes at a later stage of development. We showed statistically significant differences (p < 0.0001) in the level of intensity of Thioflavin S staining of oocytes at the early and later stages of development (Figure S1). However, Thioflavin staining is not specific enough and cannot be considered definitive evidence in identifying amyloid structures. The gold standard for the detection of amyloids is considered to be staining with Congo red and yellow-green birefringence under polarized light. In our case, no specific Congo red staining and no birefringence specific to amyloids were found in chicken oocytes (Figure 1(b,c)).

**Figure 2.**
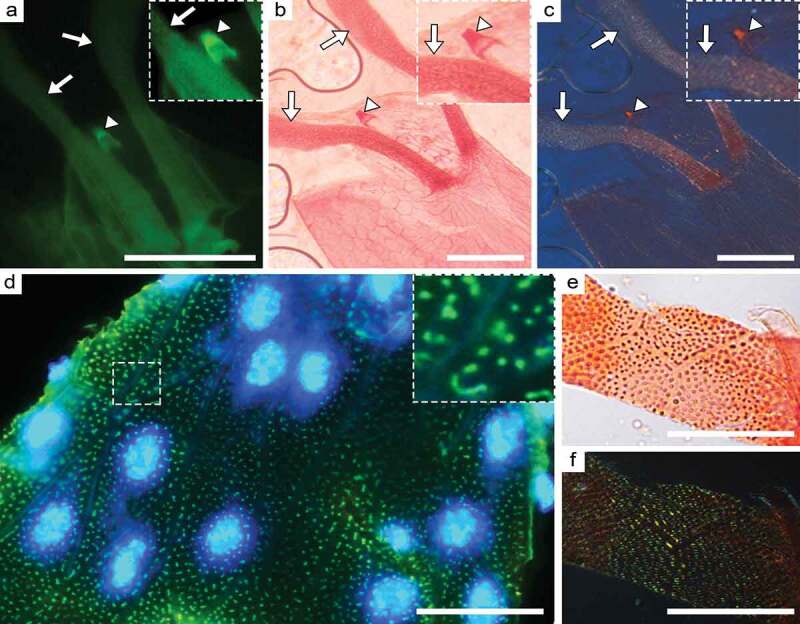
Fruit fly eggs stained with amyloid-specific dyes. The apical part of the egg stained with Thioflavin S (a) and Congo red (b, c). Slides stained with Congo red were analysed in brightfield (b) and between cross polarizers (c). The micropyle area is shown in the inset. The dorsal appendages indicated by arrows. The micropyle indicated by arrowhead. The fragment of a fruit fly egg shell stained with Thioflavin S (green fluorescence) and DAPI is shown on (d). Nuclei of degrading follicular cells are counterstained with DAPI (blue fluorescence). An enlarged fragment containing pillars stained with Thioflavin S is shown in the inset. The fragment of a fruit fly eggshell stained with Congo red and visualized in brightfield (e) and between cross polarizers (f). Scale bars 50 µm for a – c and 20 µm for (d – f)

In contrast, both Thioflavin S and Congo red specifically stain certain structures of fruit fly eggshell termed chorion. Structures such as dorsal appendages and micropyle not only stain brightly with Thioflavin S and Congo red (Figure 2(a,b)) but also exhibit birefringence in polarized light after staining with Congo red (Figure 2(c)). At high magnification it becomes evident that small structures such as pillars, which are distributed over the entire surface of the chorion, also specifically bind amyloid dyes (Figure 2(d,e)) and demonstrate a yellow-green birefringence in polarized light after staining with Congo red (Figure 2(f)). The intensity of staining of these structures with amyloid-specific dyes significantly differs from the intensity of staining of other zones of the chorion (p < 0.0001) (Figure S2). Congo red and Thioflavin equally specifically stain the structural components of the chorion of the fruit fly eggs both in unfixed and fixed material (Figure S3). Note, the micropylar apparatus is a protuberance through which a spermatozoid gains access to the oocyte membrane whereas dorsal appendages and pillars are necessary for the regulation of gas exchange. Our data clearly show that amyloid proteins are components of vital structures in fruit fly eggs.

## Discussion

In this work, we first described the structural components of chicken oocytes and fruit fly eggshell, which are stained with amyloid-specific dyes. We showed that Thioflavin S stains follicular cells and significant part of the cytoplasm of chicken oocytes, as well as some structures associated with chromatin. It was previously shown that Thioflavin stains cytoplasmic structures called Balbiani bodies in Xenopus oocytes [9]. In both chickens and Xenopus, no staining of cytoplasmic structures with Congo red dye was observed. This can be explained by the fact that Thioflavin can bind not only amyloid structures [10]. At the same time, it cannot be ruled out that the absence of Congo red staining is due to the fact, that this dye is well-suited only for the analysis of large extracellular protein deposits [11]. Currently, we can only guess which proteins are stained with Thioflavin in chicken ovaries at the early stages of development. One of the most promising candidates for this role is the main yolk precursor protein vitellogenin that is the major component of follicular cells and oocytes at the early stages of development [12]. This protein is synthesized by liver cells, forms granules, and is transported to ovarian follicular cells through blood circulation and then via transosomes to the oocyte cytoplasm [12]. In oocytes, the granules of vitellogenin are split and this protein breaks down into polypeptides, which are used as an energy source for the development of the embryo [12,13]. The hypothesis that vitellogenin or some other proteins are transported to oocytes in the form of amyloid-like granules looks attractive, but the data obtained do not allow a final conclusion.

In fruit fly eggs, both Thioflavin S and Congo red specifically stain distinct eggshell structures such as dorsal appendages, pillars, and micropyle. These extracellular structures stained with Congo red also exhibit a yellow-green birefringence, which is a characteristic feature of amyloids. All of these structures play important roles in the development of insect eggs. The respiratory appendages on either side of the dorsal midline allow eggs to carry out gas exchange when they are submerged under water [14]. The vertical pillars create cavities that facilitate gas exchange [14]. Most interestingly, micropyle, which ensures penetration of the spermatozoid into the oocyte, comprises amyloid components. As we mentioned above, the amyloid proteins are present in shells of mammalian oocytes, and there is a reason to believe that these proteins play a role in the regulation of the acrosome reaction [8]. Thus, amyloids can be involved in the regulation of the fertilization process in evolutionary distant organisms. Note that the presence of amyloids in specialized vital structures of the chorion has been shown for the first time. Dorsal appendages, pillars, and micropyle are necessary for the formation of pores in the rigid structure of the exochorion. Given the unique properties of amyloid fibrils, it can be assumed that they are an ideal biomaterial for the formation of pores in the shell of insect eggs. Silkworm eggs do not have respiratory appendages, but their shell is penetrated by many pores that are located throughout the surface [15]. The chorion of these insects contains a network of amyloid fibrils [5]. Perhaps the amyloid network is necessary for the formation of these pores. Chorionic proteins of fruit fly and silkworm have no homology, and, obviously, do not have a common origin. These data indicate that functional amyloids independently arose in the chorion of these insects. This is one of the striking examples of evolutionary parallelism in the formation of vital structures. Future studies will answer the question of whether amyloids are a universal component of insect chorion. The identification of amyloid proteins in insect chorion is also a promising task. The fruit fly eggshell contains only six major structural proteins [14], and the set of main candidates for the role of functional amyloids is very limited. Considering that *Drosophila melanogaster* is a well-studied object of genetics, the identification of a specific amyloid protein in chorion is a solvable problem.

In conclusion, our data show that amyloid proteins can be structural components of female reproductive cells of various animal species. Detection of amyloid fibrils in specialized vital structures of the fruit fly chorion opens up prospects for understanding their biological role in insect development.

## Materials and methods

### Animals

Chicken White Leghorn adult females (6-month-old) were bought from commercial stock (Federal State Unitary research farm Gene Pool (Genofond), Pushkin, Leningrad region, Russia). All procedures performed in studies involving chickens were in accordance with the ethical standards of the Ethical Committee of St. Petersburg State University (Statement #131 − 03–3 issued 01.06.2017) and Guide for the Care and Use of Laboratory Animals, 8th edition (NIH USA, 2011). Drosophila females of the Oregon R line aged 5–6 days were also used in the work. The selection of females was carried out using ether anaesthesia.

### Cytological staining

The pieces of chicken ovaries were fixed at room temperature in 4% paraformaldehyde in PBS for 4 hours, cryoprotected with 30% sucrose in PBS overnight at +4°C, frozen in Surgipath FSC 22 Frozen Section Embedding Medium (Leica Biosystems, USA) in liquid nitrogen, and stored at – 70°C. 20 μm thick cryosections were made using Leica CM1850UV cryotome (Leica Biosystems, USA) and then mounted on Superfrost-plus slides (Thermo Fisher Scientific, USA). Cryosections of chicken ovaries were stained in 1% ethanol solution of Thioflavin S (Sigma, USA). They were also subjected to nuclear staining with TO-PRO-3 (Thermo Fisher Scientific, USA). Histological staining for amyloids was also done using 1% aqueous solution of Congo red (Reanal, Hungary). The ovaries of Oregon R females were dissected in PBS. The unfixed ovaries were stained in 1% Thioflavin S solution in ethanol or in 1% Congo red aqueous solution. Nuclear staining was made using DAPI (Thermo Fisher Scientific, USA). The eggs were separated from each other, placed on a glass slide in a drop of 10% glycerol in PBS, and covered with a cover glass for further cytological analysis. The ovaries of fruit fly were also fixed at room temperature in 4% paraformaldehyde in PBS for 4 hours, cryoprotected with 30% sucrose in PBS overnight at +4°C, frozen in Surgipath FSC 22 Frozen Section Embedding Medium (Leica Biosystems, USA) in liquid nitrogen, and stored at – 70°C. The preparation of cryosections of fruit fly ovaries and their staining with amyloid-specific dyes was carried out as described above.

### Microscopy

Analysis of chicken ovaries cryosections stained with amyloid-specific dye Thioflavin S was performed using a TCS SP5 confocal microscope (Leica Microsystems, GmBH, Germany) and ‘Leica Application Suite X 3.3.0.16799’ software. The staining of fly eggs with Thioflavin S was analysed using a fluorescence Leica DM6000B microscope (Leica Microsystems GmBH, Germany) and ‘Leica QWin standart V. 3.2.0.’ software. The fluorescence was analysed using GFP and A cubes (Leica Microsystems GmBH, Germany). Cryosections of chicken ovaries and fruit fly eggs stained with Congo red were analysed in brightfield and between cross polarizers using an inverted microscope Leica DMI6000B Images were acquired using the Leica Application Suite software.

### Data analysis

Relative fluorescence intensity of Thioflavin S or Congo red was determined by ImageJ. For Thioflavin S or Congo red fluorescence intensity measurements we used images taken with a fluorescence Leica DM6000B microscope (Leica Microsystems GmBH, Germany) and ‘Leica QWin standart V. 3.2.0.’ software (GFP and N21 cubes respectively). Statistical analysis was performed using Mann Whitney test (n = 8 to 10 measurements per structure) by ‘Prism V. 9.0.0.’ software.

## Supplementary Material

Supplemental Material
