# Supplementary material for: Search for functional amyloid structures in chicken and fruit fly female reproductive cells
Source: Prion. 2020 Dec 10;14(1):278–82. doi: 10.1080/19336896.2020.1859439 (PMC7734138; doi:10.1080/19336896.2020.1859439)
Supplement: Supplemental Material [file KPRN_A_1859439_SM6206.zip › Supplementary information/Supplementary figures captures.docx]

**Supplementary figures captures**

**Figure S1.** Comparative analysis of Thioflavin S staining of chicken oocytes at early and late stages of development. **(a)** Chicken ovary cryosections were stained with amyloid-specific dye Thioflavin S (green fluorescence) and with chromatin-specific dye TO-PRO-3 (red fluorescence); “p” – primary stages of oocyte maturation (the diameter of the oocytes < 100 µm). “l” – late stages of oocyte maturation (the diameter of the oocytes > 200 µm). **(b)** Relative fluorescence intensity of Thioflavin S in primary stages of oocyte maturation in comparison to late stages is represented as mean ± SEM. Relative quantification was determined by ImageJ. Statistical analysis was performed using Mann Whitney test (* p<0.1, ** p<0.01, *** p<0.001, **** p<0.0001) by Prism. Scale bar for **a** - 100 µm.

**Figure S2.** Comparative analysis of Thioflavin S and Congo red staining of the dorsal appendages, micropyle and pillars of fruit fly chorion and chorionic zones not containing these structures. Sections **a**, **b** and **c** illustrate staining of the fruit fly chorionic pillars by Thioflavin S, while the regions without pillars do not bind this dye. The fragment of a fruit fly eggshell were stained with Thioflavin S and visualized in brightfield **(a)** and green fluorescence cube **(b, c)**. Pillars are indicated by arrows. Areas without pillars selected as a negative control are circled **(с).** Relative fluorescence intensity of Thioflavin S **(d)** or Congo red **(e)** in dorsal appendages, micropyle and pillars is represented as mean ± SEM. Relative quantification was determined by ImageJ. Statistical analysis was performed using Mann Whitney test (* p<0.1, ** p<0.01, *** p<0.001, **** p<0.0001) by Prism. Scale bars 20 µm for **a, b** and 5 µm for **c**.

**Figure S3.** Comparative analysis of Thioflavin S and Congo red staining on fixed and unfixed fruit fly chorion. The cryosection (**a, b**) and unfixed material (**c, d**) of fruit fly chorion were stained with Thioflavin S (**a, c**) and Congo red (**b, d**).
