# Supplementary figures and images for: Search for functional amyloid structures in chicken and fruit fly female reproductive cells
Source: Prion. 2020 Dec 10;14(1):278–82. doi: 10.1080/19336896.2020.1859439 (PMC7734138; doi:10.1080/19336896.2020.1859439)

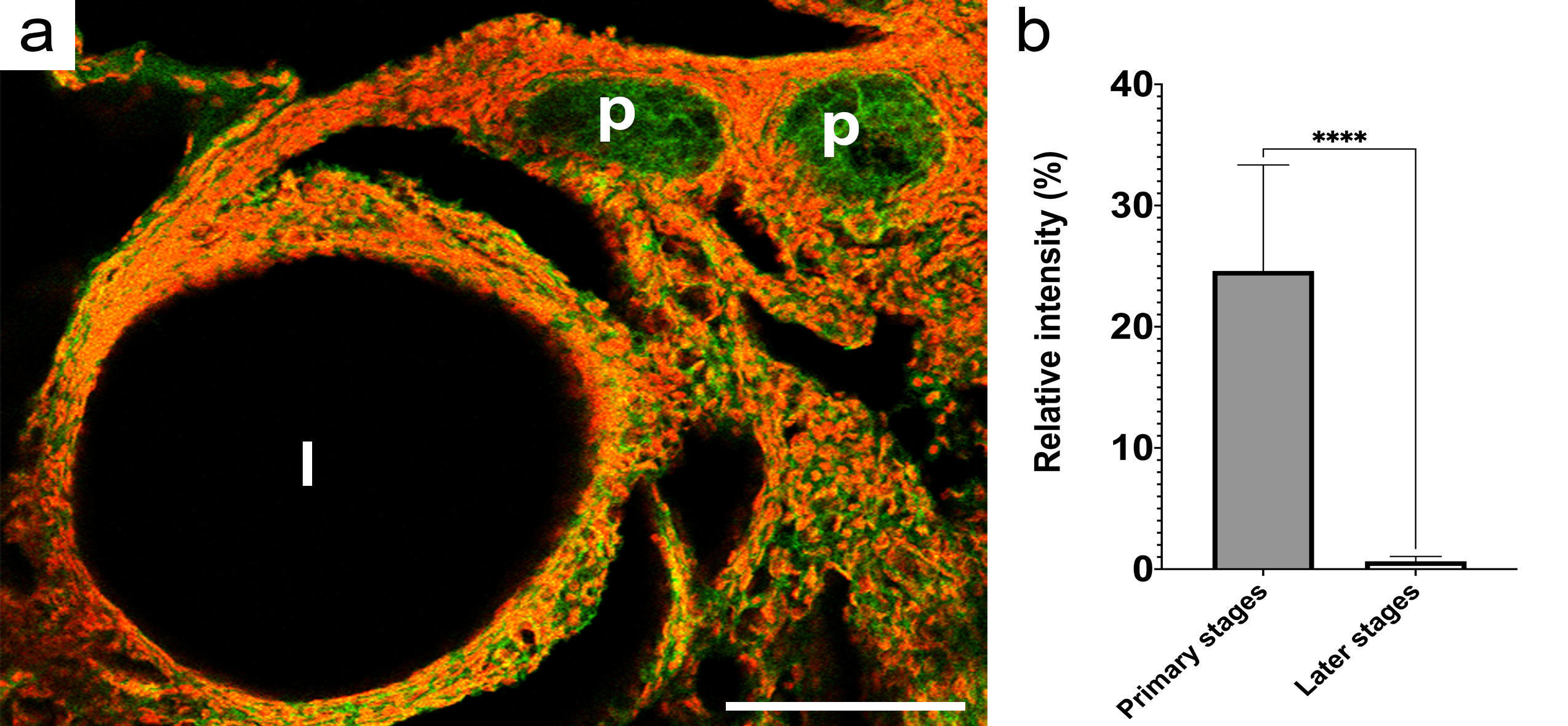

Supplement: Supplemental Material [file KPRN_A_1859439_SM6206.zip › Supplementary information/FigS1.tif]

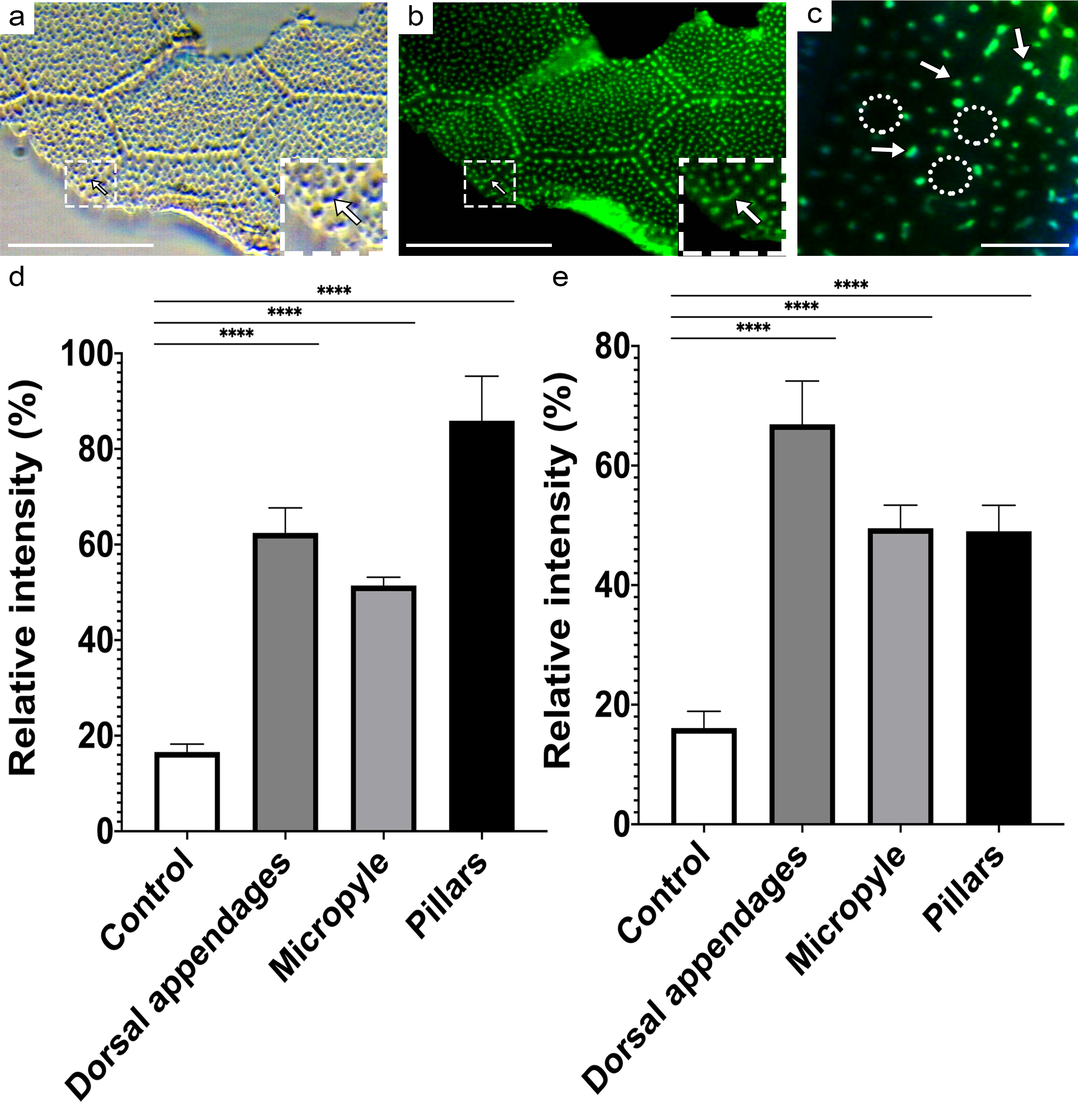

Supplement: Supplemental Material [file KPRN_A_1859439_SM6206.zip › Supplementary information/FigS2.tif]

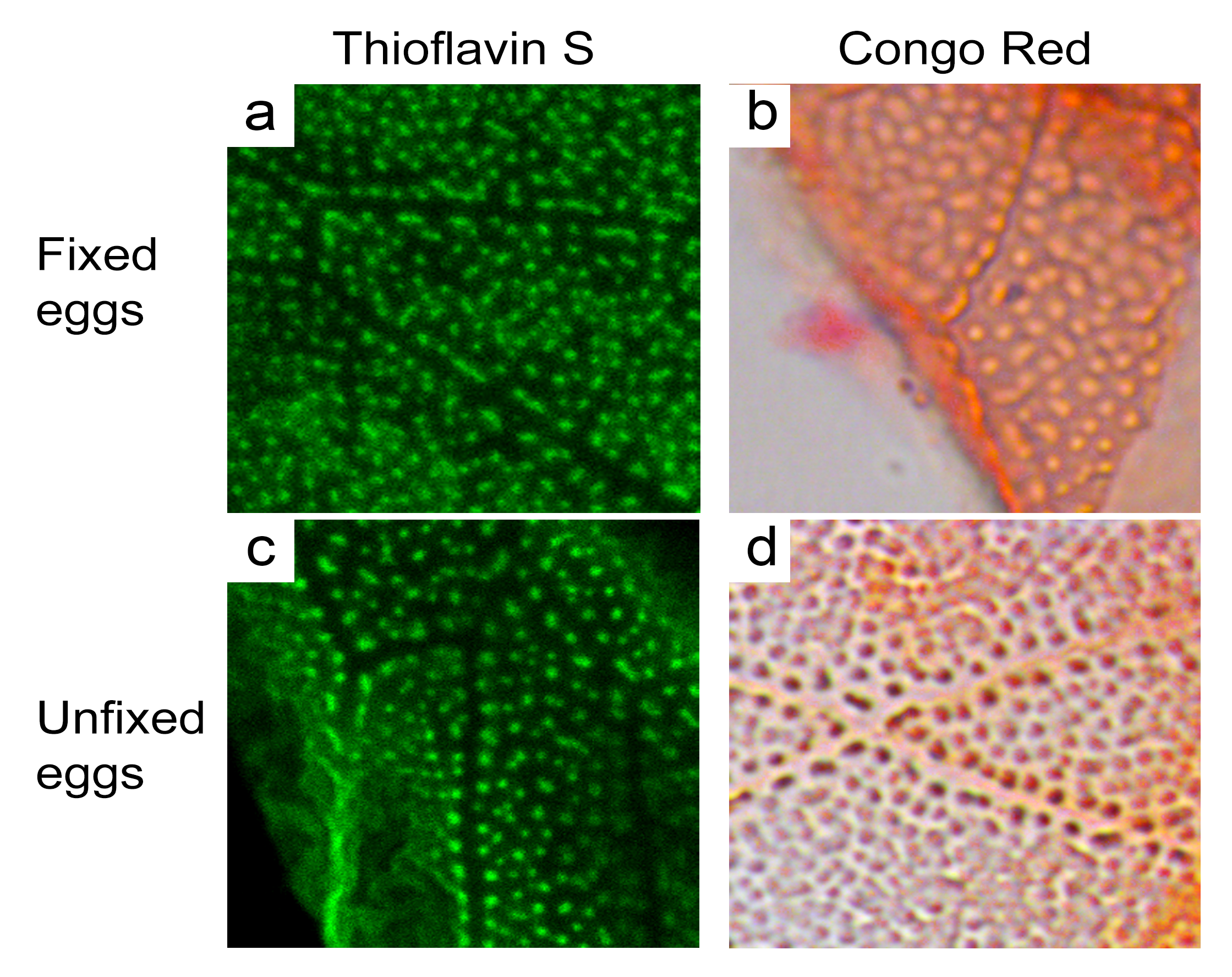

Supplement: Supplemental Material [file KPRN_A_1859439_SM6206.zip › Supplementary information/FigS3.tif]
